# Astaxanthin Protects Ochratoxin A-Induced Oxidative Stress and Apoptosis in the Heart via the Nrf2 Pathway

**DOI:** 10.1155/2020/7639109

**Published:** 2020-03-04

**Authors:** Gengyuan Cui, Lin Li, Weixiang Xu, Mingyang Wang, Danyang Jiao, Beibei Yao, Ketao Xu, Yueli Chen, Shuhua Yang, Miao Long, Peng Li, Yang Guo

**Affiliations:** ^1^Key Laboratory of Zoonosis of Liaoning Province, College of Animal Science & Veterinary Medicine, Shenyang Agricultural University, Shenyang 110866, China; ^2^Fushun Modern Agriculture and Poverty Alleviation and Development Promotion Center, Fushun 113006, China

## Abstract

This study assessed the protective mechanism of astaxanthin (ASX) against ochratoxin A- (OTA-) induced cardiac injury in mice. Four groups of mice were established: control group (0.1 mL olive oil + 0.1 mL NaHCO_2_), OTA group (0.1 mL OTA 5 mg/kg body weight), ASX group (0.1 mL ASX 100 mg/kg body weight), and ASX + OTA group (0.1 mL ASX 100 mg/kg body weight, 2 h later, 0.1 mL OTA 5 mg/kg body weight). The test period lasted for 27 days (7 days of dosing, 2 days of rest). Electrocardiogram, body weight, heart weight, tissue pathology, oxidative markers (malondialdehyde (MDA), superoxide dismutase (SOD), catalase (CAT), and glutathione (GSH)), biochemical markers (creatine kinase (CK), creatine kinase isoenzyme (CK-MB), and lactate dehydrogenase (LDH)), electron microscopy, TUNEL, and Western blot tests were used to examine the effects of OTA on myocardial injury and ASX detoxification. The results showed that OTA exposure significantly decreased both body weight and heart weight. OTA induced a decrease in heart rate in mice and decreased tissue concentrations of SOD, CAT, and GSH, while increasing serum concentrations of cardiac enzymes (CK, CK-MB, and LDH) and tissue MDA. ASX improved heart rate, cardiac enzymes, and antioxidant levels in mice. The results of tissue pathology and TUNEL assay showed that ASX protects against OTA-induced myocardial injury. In addition, Western blot results showed that the OTA group upregulated Keap1, Bax, Caspase3, and Caspase9, while it downregulated Nrf2, HO-1, and Bcl-2 protein expression. ASX played a protective role by changing the expression of Keap1, Nrf2, HO-1, Bax, Bcl-2, Caspase3, and Caspase9 proteins. These results indicate that the protective mechanism of ASX on the myocardium works through the Keap1-Nrf2 signaling pathway and mitochondria-mediated apoptosis pathway. This study provides a molecular rationale for the mechanism underlying OTA-induced myocardial injury and the protective effect of ASX on the myocardium.

## 1. Introduction

Ochratoxin A (OTA) is a toxic secondary metabolite produced by strains of Aspergillus and Penicillium and is common in food and feed products (grains, legumes, coffee, dried fruit, beer, wine, and meat) [1]. The BIOMIN Research Center indicated that both OTA and zearalenone can be detected at approximately the same level in food and feed worldwide [2]. OTA has good thermal stability, and its toxicity is not degraded within 3 h under high temperature and pressure [3]. Due to this property of OTA, humans and domestic animals are chronically affected by its toxicity, and long-term exposure to low doses of OTA has been shown to exert higher carcinogenic potential than acute exposure to high doses of OTA [2, 4, 5]. OTA is toxic for humans and animals and mainly conveys liver and kidney toxicity [6], as well as immunotoxicity [7], enterotoxicity [8], and teratogenicity [9]. It has been shown that OTA administration to rats (288.8 *μ*g/kg) by gavage led to accumulation in the lung, liver, kidney, heart, fat, intestine, and testis of rats [10]. Okutan et al. reported that low doses of OTA caused pathological damage to the rat heart as well as myocardial cell necrosis, myocardial fiber swelling, and vascular congestion [11]. Acute OTA exposure caused ultrastructural changes in the myocardium of rats [12]. It has been reported that the main cause of heart disease is the involvement of reactive oxygen species in lipid oxidation, damage of myocardial cell membranes, and dysregulation of antioxidant capacity, all of which ultimately leads to myocardial injury [13]. In addition, previous studies reported that myocardial injury causes a cascade of the caspase family, leading to apoptosis [14]. However, the mechanism of OTA toxicity, particularly if it leads to cardiac injury, remains unclear. A protective agent is required to overcome OTA-induced myocardial injury, and elucidating the toxicological mechanism of OTA on cardiac injury is of high clinical value for the prevention and treatment of myocardial injury.

Astaxanthin (ASX) is a dark red-brown fat-soluble powder, which is a potent antioxidant that belongs to the carotenoids [15]. Recent studies have demonstrated that ASX ameliorates hydroquinone-induced toxicity in retinal epithelial cells, oxidative stress, as well as iohexol-induced cytotoxicity, oxidative stress, and apoptosis in rat renal tubular epithelial cells [16, 17]. In addition, ASX offers neuroprotection against pilocarpine-induced persistent epilepsy in rats [18]. Furthermore, ASX can inhibit lipopolysaccharide-induced acute lung injury and sepsis in mice through the MAPK/NF-*κ*B signaling pathway [19]. Due to its strong antioxidant capability, ASX can be used for the protection of the heart. Xue et al. showed that ASX could inhibit oxidative stress and apoptosis in rat coronary artery microembolism- (CME-) induced cardiac chambers [20]. In addition, ASX can also decrease exercise-induced damage of the skeletal muscles and heart in mice [21]. Therefore, ASX has a promising future as an effective cardioprotective agent. Unfortunately, the ASX activity prior to OTA administration in mice has never been investigated. It remains unclear whether ASX can protect the heart from OTA damage, and potentially utilized protective mechanisms remain unclear.

This study investigated the protective effect of ASX against OTA-induced oxidative stress and apoptosis in the mouse heart to elucidate the mechanism of ASX protection at the molecular level. The results lay a clinical foundation for the role of ASX in cardioprotection as well as the prevention and treatment of OTA-induced damage.

## 2. Materials and Methods

### 2.1. Animals and Test Methods

Eighty 9-week-old C57 male mice (16-18 g) were purchased from Shandong Jinan Pengyue Laboratory Animal Breeding Co., Ltd., Shandong, China. OTA was purchased from LKT Laboratories, USA (St. Paul, MN, USA), dissolved in 0.1 mol/L sodium bicarbonate, and kept at 4°C (prepared immediately before use). ASX was purchased from Solarbio (Beijing, China), dissolved in olive oil, and stored at 4°C. Mice were acclimatized to a 12 h light/dark cycle at 25 ± 1°C and normal humidity for 3 weeks prior to the experiment. Tests were divided into four groups: (1) control group (0.1 mL olive oil + 0.1 mL sodium bicarbonate 0.1 mol/L); (2) OTA group (5 mg/kg body weight, 0.1 mL per mouse); following Hibi et al. [22] and Hood et al. [23], mice were administered with OTA (5 mg/kg body weight); (3) ASX group (100 mg/kg body weight, 0.1 mL per mouse). ASX was administered according to the test of Zhao et al. ([24]); (4) OTA+ASX group (0.1 mL of ASX 100 mg/kg body weight was administered, and after 2 h, 0.1 mL of OTA 5 mg/kg body weight was administered). The treatments were administered for 7 days and discontinued for 2 days, which was repeated for a total of 27 days. Blood samples were obtained 24 h after the end of dosing after euthanasia via anesthesia. Blood samples were allowed to stand on ice for 2 h, centrifuged at 3000 rpm for 20 min, and the supernatant serum was stored at -20°C for later use. A portion of mouse heart tissue was homogenized in 1 mL cold saline to prepare a 9% homogenate, which was centrifuged at 3000 rpm for 15 min at 4°C for biochemical analysis. The other samples were stored at -80°C to facilitate the following assays. The experimental protocol was approved by the Ethics Committee of Shenyang Agricultural University (Permit No. 264 SYXK <LIAO> 2011-0001, 20, October 2011).

### 2.2. Electrocardiographic Recorder

Electrocardiogram (ECG) recordings were performed under isoflurane anesthesia. Alligator clip electrodes were attached to the limbs of mice. Electrode gel was used to establish adequate contact. ECG was recorded by MadLab-4C/501H biomedical signal acquisition and processing system (Beijing Zhongshi Dichuang Science and Technology Development Co., Ltd., Beijing, China). All recordings were calibrated to 1 mV/10 mm with a paper speed of 50 mm/s using 5 mice per group. The electrocardiograph blindly evaluates arrhythmias in the ECG.

### 2.3. Biochemical Analysis

LDH, CK, and CK-MB kits were purchased from Changchun Huili Biotechnology Co., Ltd. (Changchun Huili Biotechnology Co., Ltd., Changchun, China). Malondialdehyde (MDA), reduced glutathione (GSH), superoxide dismutase (SOD), and catalase (CAT) were purchased from Nanjing Jiancheng Bioengineering Institute (Jiangsu, China). Serum samples were used to determine LDH, CK, and CK-MB levels. Myocardial tissue homogenate samples were used to determine MDA concentration, as well as GSH, SOD, and CAT enzyme activities. Each measurement was based on the manufacturer's description obtained from Changchun Huili Biotechnology Co., Ltd. and Nanjing Jiancheng Bioengineering Institute.

### 2.4. Histopathological Examination

From three samples per group, the myocardial ventricles of mice were excised and fixed in 10% neutral formalin, then washed in various increasing concentrations of ethanol, destained, cleared, and subsequently embedded in paraffin. Sections of 4 *μ*m thickness were stained with hematoxylin and eosin (HE) (Servicebio, Wuhan, China) and were examined by light microscopy (LEICA DM750, LEICA, Beijing, China) to assess histopathological damage.

### 2.5. Electron Microscopy

Myocardial tissue (≤1 mm^3^) was fixed in 2.5% glutaraldehyde in 0.1 M cacodylate buffer (pH 7.2-7.4) for 24 h, buffered from dimethylhydrazine to 0.1 M, and passed through a gradient of ethanol at 1% tetraoxide. The crucible was postfixed, dehydrated (50%, 70%, 95%, and 100% washing), rinsed in propylene oxide, and finally embedded in araldite mixture. Ultrathin sections were stained with uranyl acetate and lead citrate and assessed with a RiLi HT7700 (Hitachi, Japan).

### 2.6. TUNEL Assay

TUNEL analysis was performed using commercial kits according to the manufacturer's instructions. Xylene was fixed in mouse heart paraffin sections for 15 min, washed twice with absolute ethanol for 3 min each, and then washed with phosphate-buffered saline (PBS). The third wash lasted for 5 min. After the sections were fixed, 100 *μ*L (20 *μ*g/mL) of the proteinase K solution was incubated for 10 min. 10 *μ*L of 5x reaction buffer, 38 *μ*L of double-distilled H_2_O (ddH_2_O), 1 *μ*L of fluorescein isothiocyanate- (FITC-) labeled dUTP, and 1 *μ*L of terminal deoxynucleotidyl transferase (TDT) enzyme solution were mixed at room temperature and added to the incubation mixture. The samples were then strained with Dteen for 8 min. The sections were washed and photographed with a fluorescence microscope (NIKON ECLIPSE C1, NIKON, Japan).

### 2.7. Western Blotting Analysis

The total protein of 0.1 g myocardial tissue was extracted by a protein extraction kit (Solarbio, Beijing, China). After heat treatment (100°C for 5 min), protein samples were separated on a 10% sodium dodecyl sulfate- (SDS-) polyacrylamide gel and the proteins were transferred to nitrocellulose using a Trans-Blot machine (Bio-Rad). The membrane was blocked with 5% BSA in TBST (Solarbio, Beijing, China) buffer for 2 h at 25°C. Membranes were then incubated with the following primary antibodies overnight at 4°C: Keap (diluted 1 : 1000, CST, USA), Nrf2 (diluted 1 : 1000, CST, USA), HO-1 (diluted 1 : 1000, CST, USA), Bax (diluted 1 : 6000, Proteintech, Wuhan China), Bcl-2 (diluted 1 : 1500, Proteintech, Wuhan, China), Caspase3 (diluted 1 : 1000, Proteintech, Wuhan, China), Caspase9 (diluted 1 : 1000, Proteintech, Wuhan, China), and GAPDH (diluted 1 : 1000, Proteintech, Wuhan, China). GADPH was used as an internal reference to confirm equal loading. After washing with TBST buffer five times (10 min each), the membrane was incubated with the secondary antibody (diluted 1 : 3000, CST, USA) at 37°C for 1 h and then washed five times (10 min each). Proteins were detected on a DNR Bio Imaging system using the NcmECL Ultra method according to the manufacturer's instructions (Ncmbio, Suzhou, China). The Gel Quant system (Ncmbio, Suzhou, China) was used to quantify the expression of target proteins.

### 2.8. Statistical Analysis

All statistical tests were performed using SPSS 22.0 software (IBM, Almon, NY, USA), and the results were expressed as the means ± standard deviation (*X* ± SD). To analyze the difference between two groups, two-tailed Student's *t*-tests were used and significant differences among multiple groups were evaluated by one-way analysis of variance (ANOVA) and LSD methods. Differences of *P* < 0.05 were considered significant, and differences of *P* < 0.01 were considered highly significant.

## 3. Results

### 3.1. Body and Heart Weight Analysis

**Figure 1:**
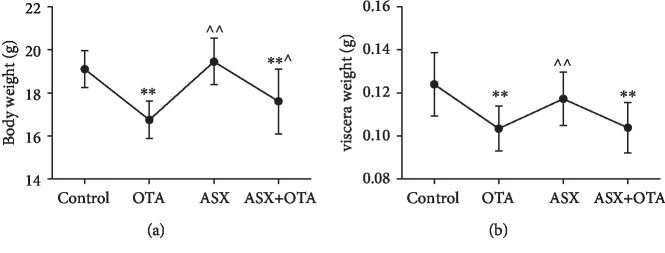
Effects of ochratoxin A (OTA, 5 mg/kg body weight), astaxanthin (ASX, 100 mg/kg body weight) and their combination on body weight and heart in mice (100 mg/kg body weight ASX+5 mg/kg body weight OTA). (a) Resulting changes in body weight of mice and (b) changes in heart weight. ^∗∗^Compared with the control group, there was a highly significant difference (*P* < 0.01). ^^^^Compared with the OTA group, there was a highly significant difference (*P* < 0.01). ^^^Compared with the OTA group, there was a significant difference (*P* < 0.05).

As shown in Figure 1(a), after 27 days of testing, mice in the OTA group and mice in the ASX+OTA group showed highly significant decreases in body weight (both *P* < 0.01) compared with the control group. As shown in Figure 1(b), the cardiac heavy pole significantly decreased in both OTA-treated mice (*P* < 0.01) and ASX+OTA mice (*P* < 0.01). However, mice in the ASX group showed a highly significant increase in body weight (*P* < 0.01) and mice in the ASX+OTA group showed a significant increase in body weight (*P* < 0.05) compared with the OTA group. Mice in the ASX group showed a highly significant increase in heart weight (*P* < 0.01). These results indicated that OTA damages the heart of mice and decreased their body weight.

### 3.2. ECG Analysis

**Figure 2:**
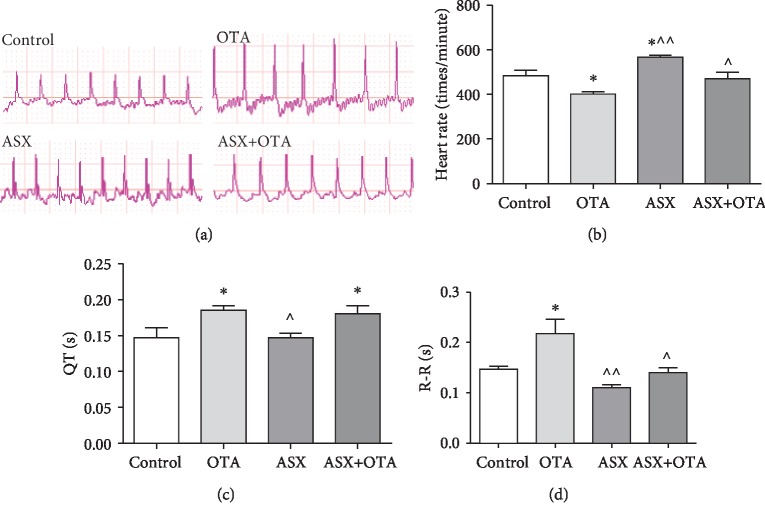
Changes in (a) electrocardiogram, (b) heart rate, (c) QT interval, and (d) RR interval. Mean ± SE of 5 mice per group. ^∗^Compared with the control group, there was significant difference (*P* < 0.05). ^^^^Compared with the OTA group, there was a highly significant difference (*P* < 0.01). ^^^Compared with the OTA group, there was a significant difference (*P* < 0.05).

As shown in Figure 2, by detecting the ECG of mice, obvious changes in heart rate could be seen. Compared with the control group, the heart rate was significantly decreased in the OTA group (*P* < 0.05) and significantly increased in the ASX group (*P* < 0.05). R-R interval and QT interval were significantly increased in the OTA group (*P* < 0.05); and the QT interval was significantly increased in the ASX+OTA group (*P* < 0.05). Compared with OTA, ASX significantly increased and decreased heart rate, R-R interval (*P* < 0.01), and QT interval (P < 0.05); ASX+OTA significantly increased heart rate (*P* < 0.05) and R-R interval (*P* < 0.05). These changes could be reversed by ASX administration. These results showed that ASX could attenuate the OTA-induced decrease of the heart rate.

### 3.3. Histopathological Observation

**Figure 3:**
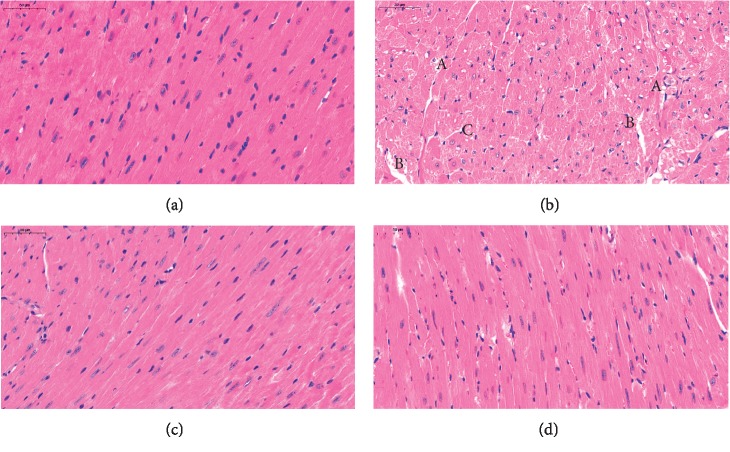
(a) Control group, (b) OTA group (5 mg/kg body weight), (c) ASX group (100 mg/kg body weight), and (d) ASX+OTA group (100 mg/kg body weight + 5 mg/kg body weight). Photographed heart tissue pathology slides (40x magnification). Mean ± SE of 3 mice per group. (a) indicates cell necrosis+vacuolar degeneration, (b) indicates fragmentation and lysis of myocardial fibers, and (c) indicates cytoplasmic vacuolar degeneration.

As shown in the pathological sections of the heart in Figure 3, the myocardial fibers of the mouse heart tissue sections of the control group (Figure 3(a)) were normal and the tissue was regularly distributed. In contrast, compared with the control phase (Figure 3(a)), in the OTA group (Figure 3(b)), mouse myocardial tissue showed extensive cytoplasmic vacuolar degeneration, myocardial swelling, fibrinolysis, small areas of hemorrhage, vascular congestion, cell swelling, and cell necrosis. The apparent recovery of myocardial tissue after ASX treatment in the ASX+OTA group (Figure 3(d)) was similar to that of the control group, without extensive vacuolar degeneration and with decreasing lytic nuclei.

### 3.4. TUNEL Analysis

**Figure 4:**
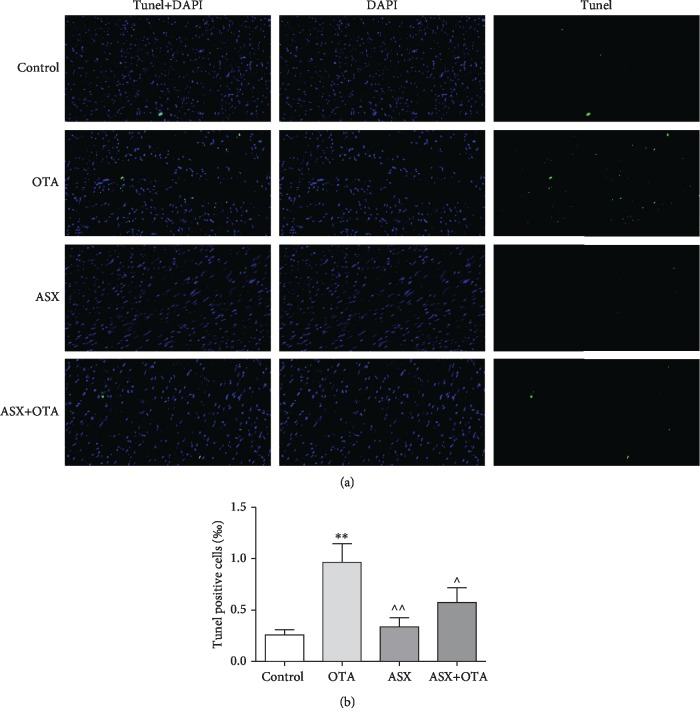
(a) TUNEL staining and (b) TUNEL-positive cell analysis. Effect of ASX on OTA-induced cardiomyocyte apoptosis. TUNEL staining (300x magnification), positive cells indicated in green; DAPI was used for nuclear staining.

The nuclei of normal cells were stained blue by TUNEL staining while apoptotic nuclei were stained green (Figure 4). Since apoptosis frequently occurs in the nucleus, superposition of blue and green fluorescence represents apoptotic cells. The number of apoptotic cells in mouse cardiomyocytes increased highly significantly in the OTA group compared with the normal group (*P* < 0.01). In contrast, the ASX group showed a highly significant decrease (*P* < 0.01) and the ASX+OTA group showed a significant decrease (*P* < 0.05) compared with OTA.

### 3.5. Electron Microscopic Analysis

**Figure 5:**
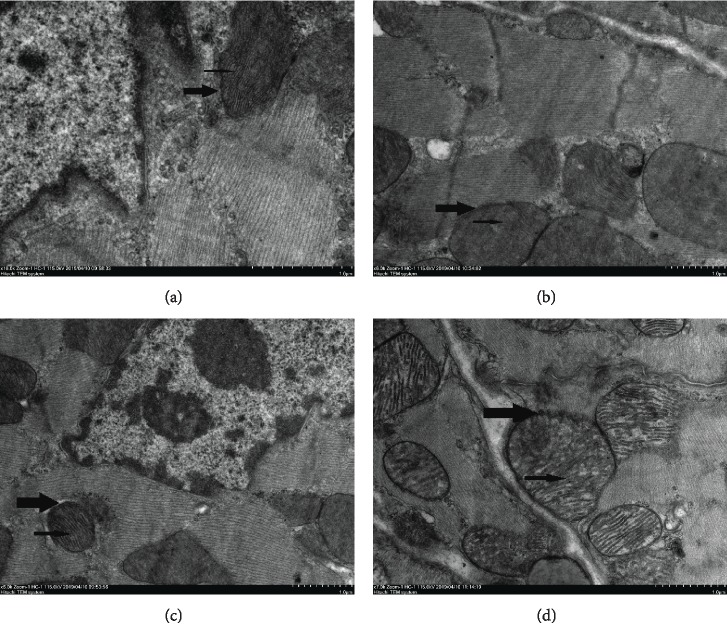
Transmission electron microscopy of cardiomyocytes. (a), (b), (c), and (d) represent control, OTA (5 mg/kg body weight), ASX (100 mg/kg body weight), and ASX+OTA (100 mg/kg body weight + 5 mg/kg body weight), respectively. Mitochondria are indicated with thick arrows and cristae with thin arrows.

As shown in Figure 5 under transmission electron microscopy, the ultrastructure and shape of the myocardium of the control group were normal: the mitochondrial structure of cardiomyocytes was normal (see Figure 5(a) thick arrow), the myocardial membrane was intact, myofilaments were arranged neatly, and mitochondria maintained a clearly integrated structure with compact cristae (see Figure 5(a) thin arrow). Compared with control, mitochondria of cardiomyocytes in the OTA group were swollen and broken (see Figure 5(b) thick arrow), and cristae dissolution disappeared (see Figure 5(b) thin arrow). Cardiomyocytes in the ASX+OTA group had normal mitochondria (see Figure 5(d) thick arrow) and intact mitochondrial cristae (see Figure 5(d) thin arrow).

### 3.6. Serum Biochemical Analysis

CK, CK-MB, and LDH were used as biochemical markers of cardiac injury. The serum of OTA-treated mice showed very significant increases of CK and CK-MB levels (*P* < 0.01) and significant increases of LDH levels (*P* < 0.05) compared with the control group. Furthermore, compared with the OTA group, the CK-MB level in the ASX group was significantly decreased (*P* < 0.01), CK and LDH were significantly decreased (*P* < 0.05), and CK, CK-MB, and LDH levels in serum of ASX + OTA mice were highly significantly decreased (*P* < 0.01). These results showed that OTA-induced cardiac injury in mice and ASX protected against cardiac injury.

### 3.7. Tissue Biochemical Analysis

**Figure 6:**
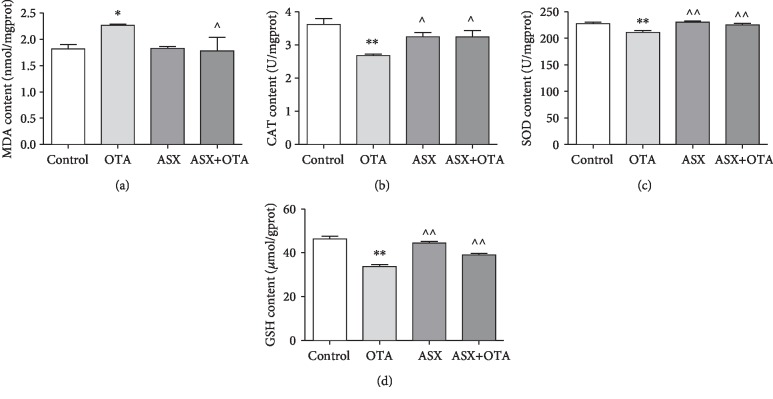
Oxidative and antioxidant parameters of tissues from each group of mice were measured. (a), (b), (c), and (d) represent changes in MDA, CAT, SOD, and GSH, respectively. ^∗^Significantly different from control (*P* < 0.05). ^∗∗^Significantly different from control (*P* < 0.01). ^^^^Significantly different from the OTA group (*P* < 0.01). ^^^Significantly different from the OTA group (*P* < 0.05).

MDA was used as the index of lipid peroxidation for cardiac injury, and GSH, SOD, and CAT were used as antioxidant markers for cardiac injury. As shown in Figure 6, compared with the control group, the OTA group showed a significant increase in MDA concentration (*P* < 0.05), a significant decrease in GSH concentration (*P* < 0.05), and a highly significant decrease in CAT and SOD concentrations (*P* < 0.01). However, compared with the OTA group, the ASX group showed a highly significant increase (*P* < 0.01) in the concentration levels of GSH and SOD, as well as a significant increase (*P* < 0.05) in the concentration level of CAT. ASX application significantly inhibited the increase in the concentration level of MDA caused by OTA and reversed the decreases of GSH, CAT, and SOD concentration levels caused by OTA. These results showed that ASX exerts a protective role against OTA-induced cardiac injury in mice.

### 3.8. Changes of Keap1-Nrf2 Signaling Pathway Proteins in the Myocardium

**Figure 7:**
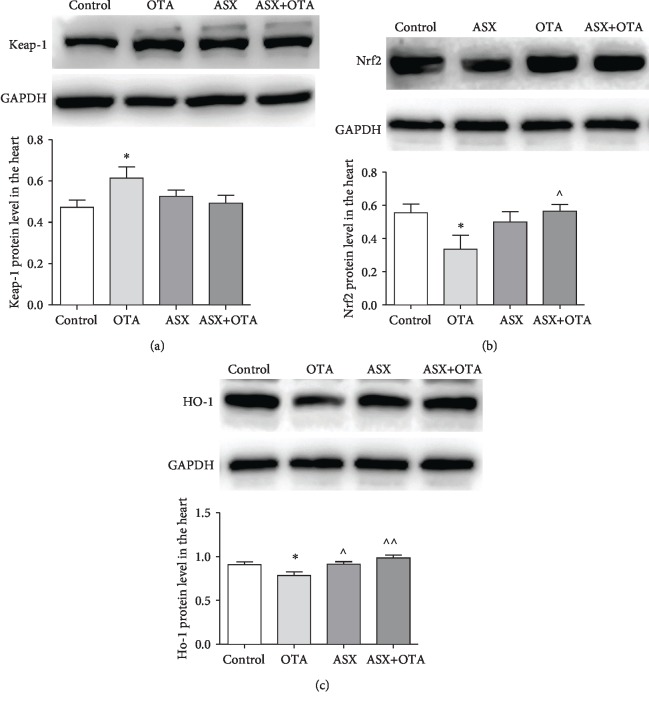
(a), (b), and (c) indicate Keap-1, Nrf2, and HO-1, respectively. Mean ± SD of 3 mice per group. ^∗^Significantly different from control (*P* < 0.05). ^∗∗^Significantly different from control (*P* < 0.01). ^^^^Significantly different from the OTA group (*P* < 0.01). ^^^Significantly different from the OTA group (*P* < 0.05).

As shown in Figure 7, compared with the control group, the protein expression of Keap1 was significantly increased (*P* < 0.05), while Nrf2 and HO-1 protein expressions were significantly decreased (*P* < 0.05) in the OTA treatment group. However, ASX alone significantly elevated HO-1 protein expression compared with the OTA treatment group (*P* < 0.01). Furthermore, Nrf2 and HO-1 protein expressions were elevated in the ASX+OTA group (*P* < 0.05 and *P* < 0.01, respectively). Keap1 protein expression was decreased but did not show a significant difference (*P* > 0.05). These results suggest that ASX plays a regulatory role in OTA-induced oxidative stress in mouse hearts.

### 3.9. Changes of Bcl-2, Bax, Caspase3, and Caspase9 Protein Expression in the Myocardium

**Figure 8:**
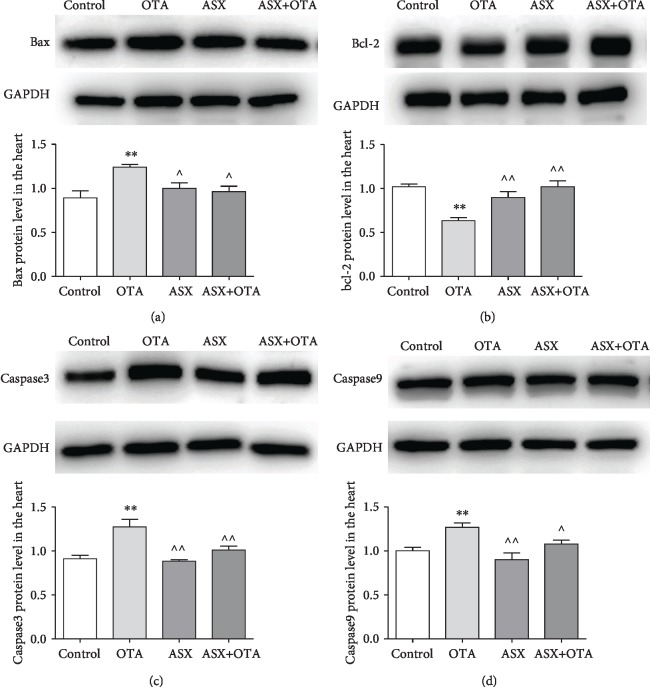
Mitochondrial apoptotic pathway protein changes were determined. (a), (b), (c), and (d) indicate Bcl-2, Bax, Caspase3, and Caspase9 protein change results, respectively. Mean ± SD of 3 mice per group. ^∗∗^Significantly different from control (*P* < 0.01). ^^^^Significantly different from the OTA group (*P* < 0.01). ^^^Significantly different from the OTA group (*P* < 0.05).

As shown in Figure 8, the results of Western blotting showed that the protein expressions of Bax, Caspase3, and Caspase9 were highly significantly increased (*P* < 0.01), while the expression of Bcl-2 was highly significantly decreased (*P* < 0.01) in the OTA group compared with the control group. However, Bax, Caspase3, and Caspase9 protein expressions decreased (*P* < 0.05) and Bcl-2 protein expression increased (*P* < 0.01) in the ASX group. Bax, Caspase3, and Caspase9 protein expressions decreased (*P* < 0.05 or *P* < 0.01) and Bcl-2 protein expression increased (*P* < 0.01) in the ASX+OTA group compared with the OTA treatment group. This illustrates that OTA causes cardiomyocyte apoptosis and that ASX is able to protect cardiomyocytes from this apoptosis.

## 4. Discussion

This study investigated the protective effect and underlying mechanism of ASX on OTA-induced myocardial injury in male mice. During the test, mice were found to have smooth and shiny fur and good overall mental state in both the control and ASX groups. However, mice in the OTA group had coarse fur, were apathetic, showed huddling behavior, and had increased urine output. Compared with the OTA group, the ASX+OTA group significantly improved the adverse status of mice. At the end of the test, OTA significantly reduced body weight and heart weight, decreased heart rate, increased QT and RR intervals, and caused abnormal ECG changes in mice, which was consistent with previous studies [11, 12, 25, 26]. This study identified pathological changes in the heart by HE staining and ultrastructural changes in the heart by transmission electron microscopy. In recent years, many researchers focused on mitochondrial dysfunction [27]. Mitochondria mainly provide energy for cells and synthesize ATP through oxidative phosphorylation on the inner mitochondrial membrane [28]. In this experiment, transmission electron microscopy showed an intact mitochondrial structure, without damage of the myocardial membrane, neatly arranged myofilaments, and intact mitochondrial cristae in the cardiomyocytes of control mice (Figure 5(a)). In contrast, the mitochondria of the cardiomyocytes of mice in the OTA group (Figure 5(b)) were swollen and damaged, and mitochondrial cristae were dissolved and had disappeared. Compared with OTA, organelles such as mitochondria were protected and the myocardium was only slightly damaged in the ASX+OTA group (Figure 5(d)) after ASX treatment. This is in line with the expectations and further confirms the successful establishment of an OTA mouse cardiotoxicity test model that can be further developed.

**Figure 9:**
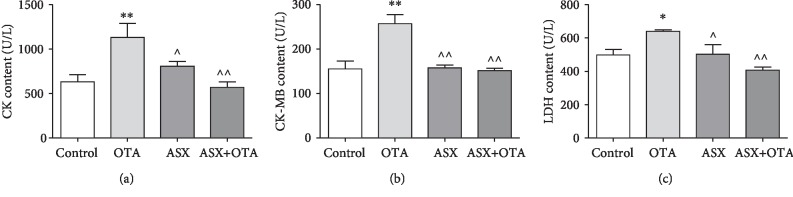
The changes of myocardial enzymes in each group of mice in serum. (a), (b), and (c) represent CK, CK-MB, and LDH, respectively. Mean ± SD of 5 mice per group. ^∗^Significantly different from control (*P* < 0.05). ^∗∗^Significantly different from control (*P* < 0.01). ^^^^Significant difference (*P* < 0.01) in comparison with the OTA group. ^^^Significant difference (*P* < 0.05) in comparison with the OTA group.

Cardiac enzymes (CK, CK-MB, and LDH) are released into the extracellular fluid at a large volume during cardiac injury; therefore, CK, CK-MB, and LDH are important monitoring indicators for myocardial injury and have been used in many cardiotoxicity tests [29, 30]. In this study, the contents of CK, CK-MB, and LDH were detected. Compared with the control group, the contents of myocardial enzymes all increased in the OTA group, while the myocardial enzymes after ASX treatment were significantly decreased (Figure 9). This indicates that ASX exerts a protective effect on OTA-induced myocardial injury in mice. Previous studies showed that mycotoxins exert toxic effects by generating excessive free radicals and lipid peroxidation [31, 32]. The present study showed a highly significant increase in MDA content in the OTA treatment group compared with the control group. Pretreatment with ASX largely inhibited the increase of MDA content. To further understand the cardiac damage induced by OTA in mice, changes in cardiac oxidative stress markers were examined in this study. This study found a significant reduction in SOD, CAT, and GSH in the OTA group compared with the control group. Compared with the OTA treatment group, the SOD, CAT, and GSH of the ASX+OTA group increased (Figure 6). These results indicate that ASX can attenuate OTA-induced cardiac oxidative damage.

Nrf2 is widely present in oxygen-consuming organs, such as the muscle tissue, heart, blood vessels, liver, kidney, and brain, and it is an important regulator of oxidative stress. Nrf2 activation can induce the synthesis of antioxidant enzymes, binomial detoxifying enzymes, and anti-inflammatory factors [33]. Under normal physiological conditions, Keap1 chelates with Nrf2 in the cytosol and thus prevents Nrf2 transcription. However, upon stimulation with various inducers such as reactive oxygen species, Keap1 in the cytoplasm dissociates from Nrf2. This dissociated free Nrf2 enters the nucleus, binds to the initiation element ARE, and induces downstream antioxidant and detoxification gene expression, including SOD, CAT, NQO1, and HO-1 [34]. This study showed that the expression of the Keap1 protein increased in the myocardium of mice in the OTA group, and the expression of total Nrf2 and HO-1 protein decreased in the myocardial tissue. Thus, OTA inhibits the activation of the Keap-1/Nrf2 pathway. Prior feeding with ASX upregulated the protein expression of total Nrf2 as well as HO-1 in myocardial tissue and decreased the protein expression of Keap-1. This reduced the oxidative stress otherwise caused by OTA. In summary, the above results indicate that ASX can improve the ability of the body to resist oxidative stress by activating the Keap-1/Nrf2 pathway. However, how ASX regulates Nrf2 transcription, translation, and binding to ARE in the nucleus requires further investigation.

Oxidative stress has been shown to cause apoptosis [35, 36], and it is well-known that apoptosis is a process of selective cell death [37, 38]. A number of mycotoxins and heavy metals can cause extensive cardiomyocyte apoptosis [31, 39, 40]. OTA can cause apoptosis in the liver and kidney, which leads to cytochrome c release [41, 42]. According to previous studies, apoptosis in the myocardium is mainly mitochondrial apoptosis. Bax and Bcl-2 are two important proteins that respond to mitochondrial apoptosis; Bax is a proapoptotic protein, and Bcl-2 is an antiapoptotic protein [38, 43]. In addition, myocardial injury in rats causes apoptosis by activating the caspase family [44–46]. This study detected cardiomyocyte apoptosis by TUNEL assay and found that OTA increased cardiomyocyte apoptosis (Figure 4). However, ASX decreased OTA-induced apoptosis in mouse cardiomyocytes. OTA significantly downregulated the Bcl-2 protein and upregulated Bax, Caspase3, and Caspase9, indicating that OTA could induce mitochondrial apoptosis in cardiomyocytes. Through pretreatment with ASX, the expression levels of cardiomyocyte apoptotic proteins such as Bax, Bcl-2, Caspase3, and Caspase9 changed to enhance myocardial antiapoptosis ability. Combined with cardiac TUNEL results, this demonstrated the protective effect of ASX on OTA-induced myocardial mitochondrial apoptosis.

## 5. Conclusions

In conclusion, this study experimentally demonstrated that ASX can protect against oxidative stress and mitochondrial apoptosis caused by OTA exposure in the hearts of mice. The cardioprotective effects of ASX may be mediated by the modulation of the Nrf2 pathway and the mitochondrial apoptotic pathway.

## Data Availability

The data used to support the findings of this study are included within the article.
